# Move your body, stay away from depression: a systematic review and meta-analysis of exercise-based prevention of depression in middle-aged and older adults

**DOI:** 10.3389/fpubh.2025.1554195

**Published:** 2025-03-07

**Authors:** Xiao-Dong Zhao, Sae-Sook Oh, Zheng Zhang, Chong Wang

**Affiliations:** ^1^College of Sports Science, Kyonggi University, Suwon, Gyeonggi, Republic of Korea; ^2^College of Sports Science, Northwest Normal University, Lanzhou, Gansu, China

**Keywords:** depression, older adult, exercise, prevent, meta-analysis

## Abstract

**Objective:**

This study aimed to systematically assess the efficacy of exercise interventions in preventing depression among middle-aged and older adults. It further sought to dissect the impact of various intervention factors on the exercise-depression nexus within this demographic.

**Methods:**

We conducted a comprehensive search across multiple databases including PubMed, Embase, Web of Science, and the Cochrane Library, spanning from their inception through April 2024. The initial search yielded several studies, from which 11 papers involving a total of 792 subjects were selected based on predetermined inclusion criteria.

**Results:**

Our meta-analysis revealed a significant overall effect size (SMD = −3.64) with a 95% confidence interval of [−4.81, −2.48] and a *p*-value of <0.00001, underscoring the potent preventative impact of exercise on depressive symptoms in the target population. However, the studies exhibited substantial heterogeneity (*I*^2^ = 87%).

**Conclusion:**

Exercise interventions, particularly balance and gentle training as well as functional training, significantly reduce the risk of depression among middle-aged and older adults. The most pronounced effects were observed in group exercise settings and with exercise sessions lasting 30–40 min. Interventions of medium duration generally outperformed shorter-term interventions. Despite these promising results, the observed heterogeneity and other potential study limitations suggest a need for further research involving larger samples and more comprehensive study designs to refine and optimize exercise protocols for depression prevention in this population.

## 1 Background

A significant shift is occurring across the globe, particularly pronounced in developed regions: the aging of the population (1). A World Health Organization report forecasts that the segment of the population aged 65 and over will rise from 9% in 2020 to 16% by 2050 (2). This demographic shift brings with it increasing concerns about the mental health of middle-aged and older adults, which is emerging as a critical area of public health focus. The transition into middle and old age represents a crucial juncture in life, characterized by retirement, deteriorating health, and the loss of close family and friends. These life events are known catalysts for mental health challenges, especially depression (3). Furthermore, societal changes such as the trend toward smaller, nuclear families diminish social engagement opportunities for the older adult, thereby enhancing feelings of loneliness and social exclusion (4). This sense of isolation, coupled with a lack of robust social support networks, significantly contributes to mental health issues within this age group.

Depression, a prevalent mental health concern, exhibits heightened complexity and risk within the middle-aged and older demographic. Its prevalence and severity are frequently underestimated, as individuals in these age groups may conceal their emotional struggles or dismiss them as a normal aspect of aging (5). Moreover, depression can exacerbate other medical conditions, notably cognitive impairments and dementia, with a marked acceleration in cognitive decline observed in older adults (6). Depression is further linked to an array of physical health issues, especially cardiovascular diseases (7). Research indicates that individuals with depression face a risk of cardiovascular disease nearly twice that of the general population, a disparity partly attributed to reduced motivation for regular physical activity. Additionally, depression can directly impact physical functions, including heart rate and blood pressure regulation (8). The implications of depression extend beyond the individual, significantly straining medical and social resources. Middle-aged and older adults suffering from depression are more likely to require comprehensive medical care, encompassing both mental health services and long-term care facilities (9, 10). This increased demand for services not only affects the individuals and their families but also places a substantial burden on the healthcare system overall. Furthermore, the financial impact of depression is considerable, encompassing treatment costs, loss of income due to disability, and the extensive time and energy devoted by caregivers (11).

Exercise has been validated as an efficacious non-pharmacological intervention for both preventing and alleviating symptoms of depression among middle-aged and older individuals (12). The benefits of physical activity extend beyond mere symptom management, enhancing mental health and emotional well-being through various biological and psychological pathways. These include the modulation of neurotransmitters, enhancement of endocrine function, and bolstering of social interactions and self-efficacy (13, 14). Unlike pharmacological treatments, exercise is devoid of the adverse side effects commonly associated with medications. Furthermore, it confers additional health advantages, notably improving cardiovascular health, muscle strength, and bone density. Thus, regular physical activity is not only instrumental in warding off depression among older adults but also plays a pivotal role in promoting overall health and vitality (15).

Current research into the therapeutic effects of exercise on depression among middle-aged and older adults is burgeoning, with numerous studies substantiating the benefits of regular physical activity in this demographic. These investigations primarily focus on the short-term impacts of exercise interventions on enhancing the psychological well-being of patients, such as reducing anxiety, uplifting mood, and improving social functioning (16–18).

For instance, Andreas Heissel’s research advocates for exercise as an efficacious treatment for depression and depressive symptoms, recommending evidence-based approaches including supervised, moderate-intensity group exercise and aerobic programs (19). Similarly, Yumeng Xie’s findings underscore that moderate-intensity exercise suffices to alleviate depressive symptoms, though higher doses may further enhance overall functioning. Xie also highlights the benefits of integrating aerobic with mind–body exercises (20). Complementing these, Lange KW’s research suggests that multimodal exercise—combining various types of physical activities—might be the most effective strategy for mitigating depressive symptoms, proposing that a diverse exercise regimen could yield superior improvements in depression (21).

However, it is important to note that while there is a substantial body of literature focused on exercise as a treatment for depression, comparatively less research has been dedicated to its preventive capabilities in middle-aged and older adults. This gap underscores the need for further studies to explore and validate exercise’s prophylactic potential in this context. The methodology underlying prevention trials poses significant challenges, particularly when assessing the efficacy of exercise in forestalling the onset of depression among middle-aged and older adults. Such trials necessitate longitudinal approaches, requiring participants to be monitored over extended periods to accurately gauge the preventive impact of physical activity. This longitudinal monitoring demands not only longer timescales but also more intricate study designs to ensure reliable results.

Moreover, prevention trials ideally target the general population that has not yet exhibited significant depressive symptoms, which introduces additional complexities in both recruitment and data interpretation. Recruiting a sufficiently large and representative sample from this demographic can be challenging, as it involves individuals at potential risk but who have not yet manifested clear signs of depression. Furthermore, the absence of initial symptoms complicates the task of clearly demonstrating the preventive benefits of exercise, as changes in mental health status must be meticulously tracked and attributed to the intervention amidst various potential confounding factors.

Despite challenges, some existing prevention studies offer valuable insights into the potential role of exercise in forestalling depression across various age groups. For instance, Mandy X. Hu’s research indicates that physical activity can act as a preventative measure against depression in the general population, although evidence specifically regarding its preventive impact prior to any onset of depressive symptoms remains scant (22). Additionally, U Hemmeter’s findings suggest that regular exercise significantly lowers the risk of developing depression in older adults, highlighting its potential as a protective strategy (23).

However, the reliability of these studies is contingent upon their quality and methodological rigor. According to the AMSTAR 2 scoring system, many relevant meta-analyses have been rated as moderate to low quality, which raises concerns about the selection of studies, data extraction processes, and assessments of bias risks (24). These methodological shortcomings can obscure the true effectiveness of exercise as a preventive intervention for depression, potentially affecting both our understanding and the broader dissemination of these findings.

To bridge this knowledge gap, more systematic and rigorously designed studies are necessary. Future research should focus on exploring how various types and intensities of exercise influence depression prevention among middle-aged and older adults. Such studies will enhance our comprehension of the mechanisms through which exercise serves as an effective non-pharmacological approach to preventing depression in these populations, thereby informing better public health strategies and interventions.

## 2 Methods

This meta-analysis was performed according to the Preferred Reporting Items for Systematic Reviews and Meta-Analysis statement and the Cochrane Collaboration Handbook. The protocol was registered on PROSPERO (CRD42024546184).

### 2.1 Data sources and searches

The systematic search was conducted by two independent reviewers (ZXD and ZZ) in four databases: the Cochrane Library, Embase, PubMed and Web of Science, and was designed to retrieve articles up to April 2024, with disagreements resolved by consensus and by a third reviewer (WC) in case of disagreement. Terms from the Medical Subject Headings (MeSH) and words from the text were used as follows: (“Elderly” OR “Elderly” OR “Old” OR “Senior” OR “prenatal” OR “Mature” OR “Older” OR “Elderly person” OR “Aged person” OR “Senior citizen” OR “Elderly individual” OR “Advanced in years” OR “Geriatric” OR “Over the hill” OR “Elderly people” OR “Older generation” OR “Senior person”) AND (“Depression” OR “Depressive Symptoms” OR “Depressive Symptom” OR “Symptom, Depressive” OR “Emotional Depression” OR “Depression, Emotional”) AND (“Exercise” OR “Exercises” OR “Physical Activity” OR “Activities, Physical” OR “Activity, Physical” OR “Physical Activities” OR “Exercise, Physical” OR “Exercises, Physical” OR “Physical Exercise” OR “Physical Exercises” OR “Acute Exercise” OR “Acute Exercises” OR “Exercise, Acute” OR “Exercises, Acute” OR “Exercise, Isometric” OR “Exercises, Isometric” OR “Isometric Exercises” OR “Isometric Exercise” OR “Exercise, Aerobic” OR “Aerobic Exercise” OR “Aerobic Exercises” OR “Exercises, Aerobic” OR “Exercise Training” OR “Exercise Trainings” OR “Training, Exercise” OR “Trainings, Exercise”). Specific details of the search algorithms for each database are provided in Supplementary Material 1.

### 2.2 Inclusion and exclusion

Following the PICOS (Participants, Intervention, Comparison, Outcomes, Study design) framework recommended by Cochrane for systematic reviews, we defined our study’s inclusion criteria as follows: Participants (P) are middle-aged and older adult individuals, aged 45 years and older. The intervention (I) involves multifaceted exercise programs varying in content, intensity, duration, frequency, and cycles. Comparisons (C) are drawn against usual care or placebo. Outcomes (O) primary focus on depressive symptoms, quantified using standardized instruments such as the Hospital Anxiety and Depression Scale (HADS), the Geriatric Depression Scale (GDS), and Beck’s Depression Inventory (BDI), among others. Secondary outcomes consider factors including quality of life, sleep quality, cognitive function, and more. The methodology (S) primarily encompasses randomized controlled trials (RCTs), which are considered the gold standard in clinical effectiveness research. Exclusion criteria are defined to omit studies involving participants with psychiatric disorders, those incapable of physical activity, interventions focusing on pharmacological treatments, psychotherapies, or non-exercise modalities, non-original studies such as commentaries, case reports, and reviews, studies with methodological flaws such as inadequate sample sizes, absence of control groups, or unclear randomization protocols, studies not published in English, and non-RCT studies.

### 2.3 Assessment of risks of bias

In adherence to the esteemed Cochrane Collaboration guidelines, reviewers ZXD and ZZ independently conducted a meticulous risk of bias assessment for each study included in our analysis. The Cochrane Collaboration’s guidelines represent a comprehensive and universally recognized framework, enabling a standardized evaluation of the methodological integrity of each study. This detailed assessment process, ingrained in the Cochrane Collaboration ethos, involves a granular examination of potential biases. Key areas scrutinized include randomization processes, allocation concealment, the blinding of participants and researchers (notably challenging in certain ethical contexts), completeness of outcome data, risks of selective reporting, and other potential biases. Each study’s evaluation was independently undertaken by the reviewers, with discrepancies resolved through discussion or, when necessary, by consulting a third reviewer (WC). The outcomes of this rigorous bias risk assessment are meticulously compiled in a risk of bias table, which is available in Supplementary Material 2. This table provides a transparent and comprehensive overview of the methodological quality of each study considered. Importantly, when interpreting the results of the meta-analyses and forming conclusions regarding the efficacy of exercise preventions for depression in middle-aged and older adults, these bias risk assessments were carefully factored into our deliberations. This approach ensures a balanced and methodologically sound interpretation of the data, upholding the high standards of evidence synthesis championed by the Cochrane Collaboration.

### 2.4 Data extraction

With a standardized form, two reviewers (ZXD and ZZ) independently extracted the pertinent data from each included study, encompassing essential details such as author names, year of publication, sample size of the intervention and control groups, age group characteristics of both intervention and control groups, type of intervention, intervention length, frequency, and duration, type of control group, and outcome measures. The utilization of a standardized form ensured consistency and accuracy in data extraction across all studies, minimizing the risk of errors and enhancing the reliability of the collected information. Each reviewer diligently recorded the required data elements from the eligible studies, and any discrepancies or uncertainties were resolved through discussion or consultation with a third reviewer (WC) if necessary. By employing this rigorous and systematic data extraction approach, we obtained comprehensive and reliable information from the included studies, forming the foundation for our comprehensive META analysis. The detailed data extracted from each study are presented in the supplementary materials, providing transparency and facilitating a thorough understanding of the primary characteristics of the studies included in our research.

### 2.5 Assessment of overall effect size

Statistical analyses were conducted using Review Manager V.5.3, and overall effect sizes were calculated based on the statistical analyses of the results from the measurement scale tests of the 12 included articles. Hedge’s g standardized effect sizes were utilized for each included study to measure the intervention’s effect size, with effect sizes of 0.2, 0.4, and 0.8 indicating small, medium, and large effects, respectively. To ensure consistency and that all effect sizes were in the expected direction of the intervention, *p* < 0.05 was considered significant. Given that there are different measures of the effect of exercise on depression in middle-aged and older adults, the standardized mean difference (SMD) was chosen as it reflects the overall intervention effect size. To synthesize the effect of physical activity on depression scores in middle-aged and older adults in the meta-analysis, the standardized mean difference (SMD) was calculated using the Practical Meta-Analysis Effect Size Calculator (Wilson) along with its corresponding 95% confidence intervals. A heterogeneity test was also conducted to assess the extent of differences between the included studies in describing the overall effect sizes. Heterogeneity was assessed using methods such as the Q statistic and the I^2^ indicator. *I*^2^ values quantitatively assessed heterogeneity, where 0% indicated no heterogeneity, ≥25% indicated low heterogeneity, ≥50% indicated moderate heterogeneity, and ≥ 75% indicated high heterogeneity. When *I*^2^ values indicated moderate to high heterogeneity, a random-effects model was used for data combination, and conversely, a fixed-effects model was utilized.

### 2.6 Subgroup analysis of exercise intervention programmes

In this study, subgroup analyses were meticulously structured to elucidate potential sources of heterogeneity and to dissect the impacts of various exercise interventions on depression prevention in middle-aged and older adults. These analyses aim to pinpoint the specific exercise modalities that most effectively stave off depression, thereby furnishing more targeted recommendations for clinical practice.

Subgroup analyses will focus on several key domains:

1. Individual versus group exercise: This comparison aims to clarify the differential impacts of solitary versus communal exercise settings on depression prevention in the target demographic.
2. Classification of exercise forms: Exercise modalities were categorized into four principal types: aerobic training, resistance training, balance and flexibility training, and functional training.
3. Exercise duration and total exercise time: These analyses will explore how varying lengths of exercise sessions and total intervention durations (measured in weeks or months) influence the efficacy of depression prevention.

For each subgroup, data were meticulously extracted from included studies. We utilized Review Manager V.5.3 to compute effect sizes (Hedge’s g) and their 95% confidence intervals, assessing statistical significance at a threshold of *p* < 0.05. Heterogeneity tests determined the presence of significant discrepancies between subgroups (25).

The outcomes of these subgroup analyses will deepen our comprehension of how specific exercise protocols mitigate depression in middle-aged and older adults, offering nuanced insights that can guide clinical applications. Additionally, these findings will be extensively discussed in the discussion section of our paper, providing valuable directions for future research and practical implementations.

## 3 Results

### 3.1 Search process

**Figure 1:**
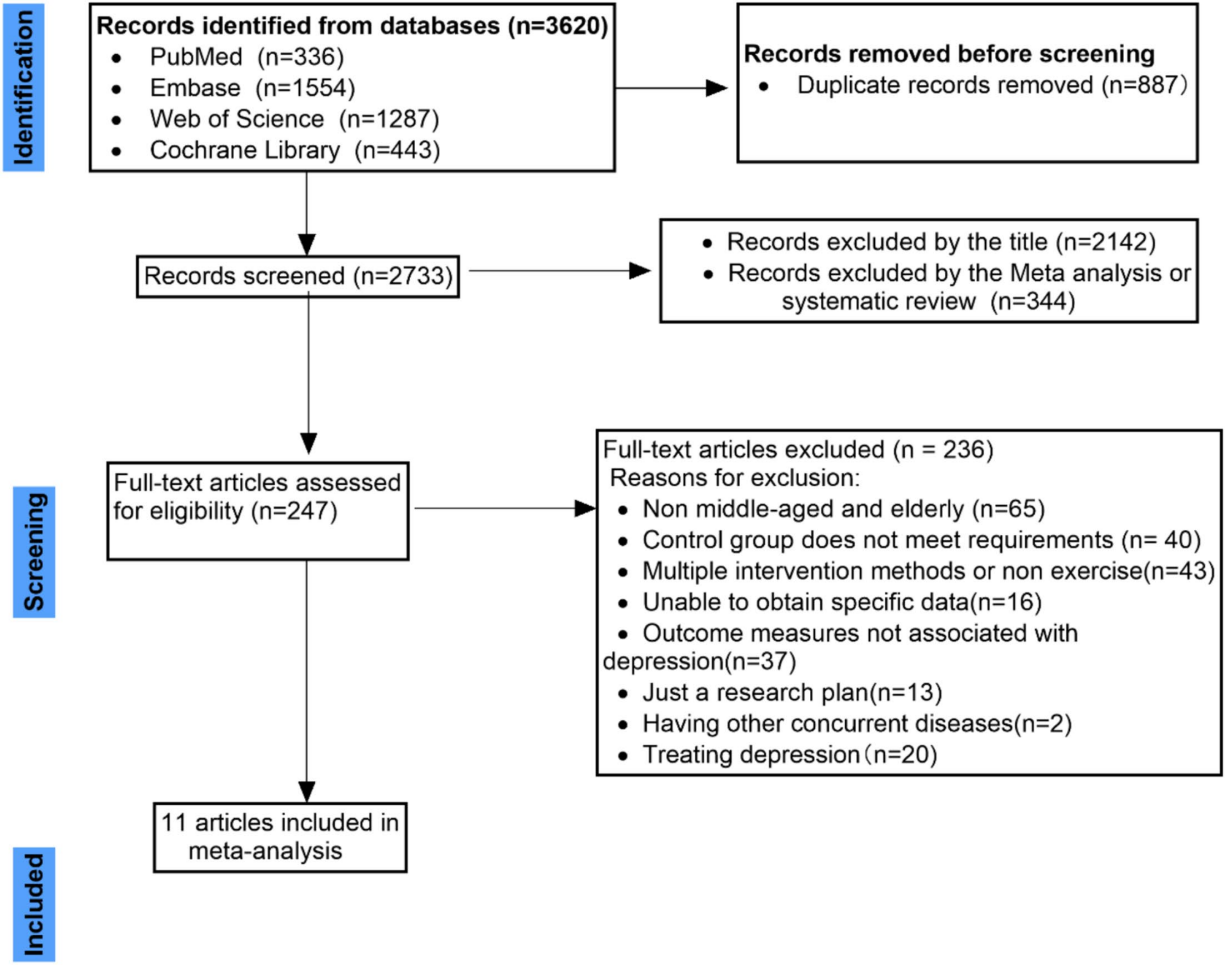
Flowchart and selection of studies.

In our comprehensive literature search, we queried four distinct databases, initially identifying 3,620 trials. Subsequent to the removal of duplicate entries, a total of 2,733 trials were excluded from consideration for meta-analysis and systematic review. This exclusion was based on an initial screening of titles and abstracts, which narrowed the pool to 247 trials necessitating full-text evaluations. Upon detailed examination, 236 of these trials were further excluded due to several disqualifying factors: non-involvement of the target demographic of middle-aged and older adult populations, non-compliance with predefined intervention protocols, failure to meet specified outcome criteria, or the primary treatment focus on depression rather than prevention. Ultimately, the meta-analysis proceeded with data extracted from 11 qualifying studies. Each step of this rigorous selection process is meticulously documented in Figure 1, which illustrates the filtering criteria and the progressive narrowing of the study sample.

### 3.2 Characteristics of the included studies and participants

Table 1 presents the characteristics of the 11 studies incorporated into this meta-analysis. These studies, published between 2005 and 2023, varied significantly in sample sizes, ranging from 16 to 160 participants. Detailed demographics such as sample sizes of intervention and control groups, mean age, and gender distribution across each study are systematically outlined in Table 1. The interventions investigated include a diverse array of exercise types: yoga, tai chi, walking, qigong, Pilates, and resistance training. The duration of these exercise interventions spanned from 2 to 18 months, with the frequency of sessions ranging from once weekly to four times weekly. The outcomes measured were primarily focused on depression, utilizing various scales to assess its severity and impact. These included the Geriatric Depression Scale (GDS), Depression Anxiety Stress Scale-21 (DASS-21), Hospital Anxiety and Depression Scale (HADS), Profile of Mood States (POMS), Hamilton Depression Scale (HAMD), Beck Depression Inventory (BDI), and the Goldberg Anxiety and Depression Scale (GADS).

**Table 1:** Characteristics of the included studies and participants.

Studies	Sample size (IG/CG)	Age range (IG/CG)	Gender ratio (IG/CG) M:F	IG type	CG type	Frequency/duration	Outcome measures
Silvano Zanuso (2012)	10:10	≥ 65		strength training	usual care	3 times weekly/12 weeks	POMS
Hanna Karen Moreira Antunes (2005)	23:23	68.08 ± 5.49/65.86 ± 3.80	23:0/23:0	aerobic fitness	usual activities	3 times weekly/6 months	GDS
María del Carmen Carcelén-Fraile (2022)	57:60	69.70 ± 6.15/69.75 ± 6.76	0:57/0:60	Qigong training	usual activities	2 times weekly/12 weeks	HADS
José Alberto Laredo-Aguilera (2018)	20:18	75.44 ± 5.31/76.35 ± 6.45	6:32	functional training	usual activities	3 times weekly/10 weeks	GDS
Walid Bouaziz (2018)	30:30	72.9 ± 2.5/74.3 ± 3.4	9:21/7:23	short-term Interval Aerobic Training	usual activities	2 times weekly /9.5 weeks	GADS
Souad Baklouti (2023)	65:95	≥ 65	42:23/56:39	web-based Hatha yoga	usual activities	2 times weekly/2 months	DASS-21
Kiwol Sung (2009)	9:7	69.6 ± 4.4/71.6 ± 2.3	0:9/0:6	functional exercise	usual activities	3 times weekly /16 weeks	GDS
P. Bernard (2014)	61:60	65.46 ± 4.37/65.5 ± 4.03	0:61/0:60	Walking	usual activities	3 times weekly/6 months	BDI
Agustín Aibar-Almazán (2019)	55:52	69.98 ± 7.83/66.79 ± 10.14	0:55/0:52	Pilates training	usual activities	2 times weekly/12 weeks	HADS
Tiia Kekäläinen (2017)	26:23	68.9 ± 2.7/68.3 ± 2.3	12:14/13:10	resistance training	usual activities	1 times weekly/9 months	BDI-II
Xinan Zhang (2014)	28:30	65.50 ± 5.54/64.10 ± 4.36	13:15/16:14	Tai Chi	usual activities	4 times weekly/18 months	HAMD

IG, Intervention Group; CG, Control Group; BDI, Beck Depression Inventory; HADS, Hospital Anxiety Depression Scale; GDS, Geriatric Depression Scale; GADS, The Goldberg Anxiety and Depression Scale; DASS-21, Depression Anxiety Stress Scale-21; POMS, Profile of Mood States; HAMD, Hamilton Depression Scale.

### 3.3 Risks of bias

Among the 11 studies included in our meta-analysis, all demonstrated low risk in random sequence generation. Seven studies showed low risk in the concealment of allocation, while four were assessed as having an unknown risk due to underreporting. Notably, participant blinding was consistently problematic, with all studies presenting a high risk in this area. Assessment of bias in blinding the outcome assessors revealed three studies at low risk, with the remaining eight at an unknown risk due to underreporting.

Regarding the integrity of the outcome data, 11 studies were rated low risk for incomplete outcome data, reflecting robust data handling protocols. However, selective reporting of results was another concern: ten studies were deemed low risk, but one was classified at unknown risk, suggesting potential issues in reporting transparency. Additionally, all studies were marked as having an unknown risk for other biases due to insufficient information, which may compromise the reliability of findings. The comprehensive assessment of risk of bias for each study is detailed in Supplementary Material 2, providing a critical framework for interpreting the studies’ contributions to the field.

### 3.4 Meta-analysis

#### 3.4.1 Baseline period test

In this study, rigorous analyses were conducted on the baseline data of the experimental and control groups across the 11 included studies to ascertain comparability prior to intervention onset. The integration of baseline data aimed to determine any pre-existing differences in depressive symptoms between groups. Given the diversity of assessment tools used across studies—including the Beck Depression Inventory-II (BDI-II), Geriatric Depression Scale (GDS), Hospital Anxiety and Depression Scale (HADS), and Profile of Mood States (POMS)—the Standardized Mean Difference (SMD) was employed as a harmonizing statistic to overcome scale discrepancies.

**Figure 2:**
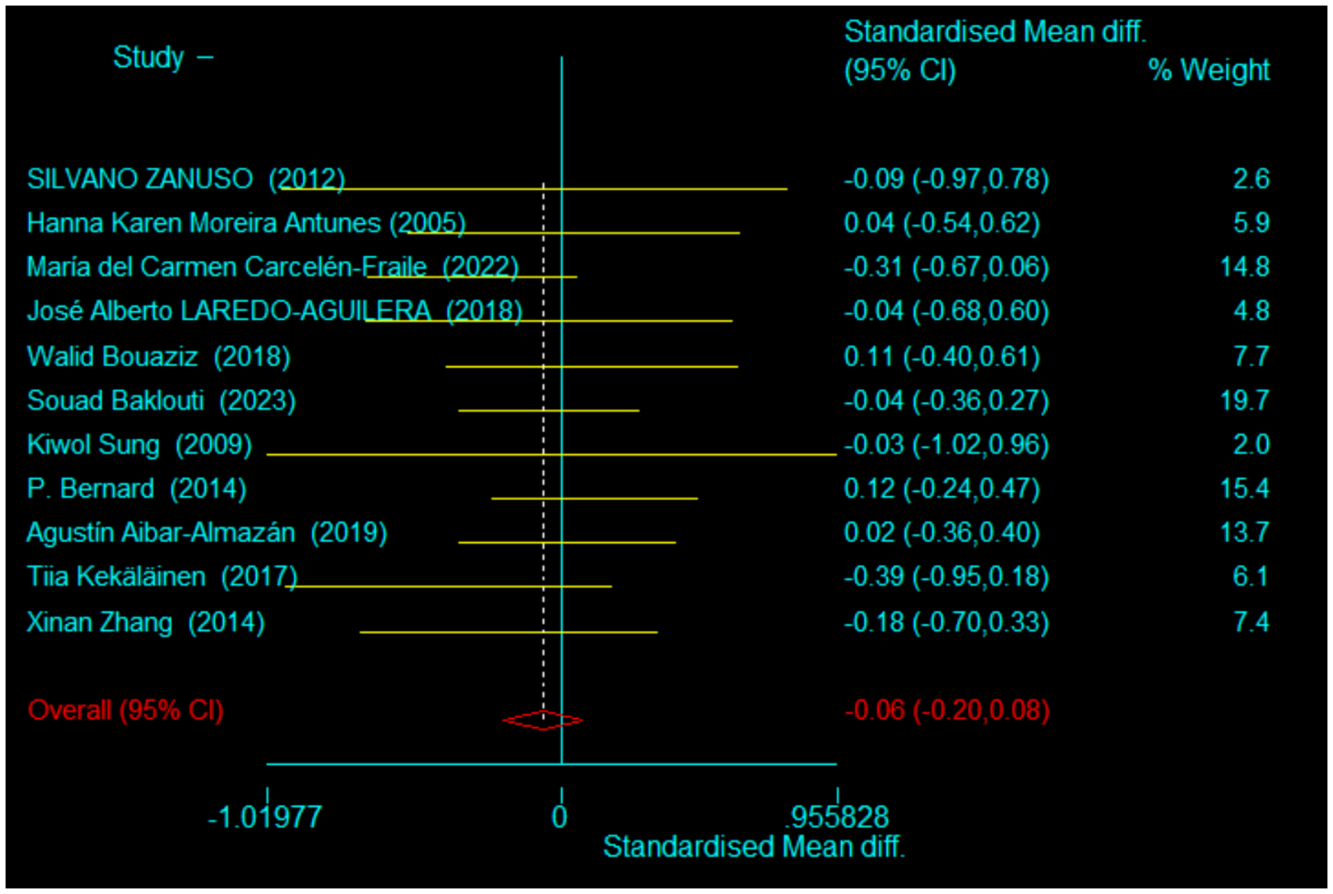
Baseline period test.

The results of our baseline meta-analysis, detailed in Figure 2, demonstrated a pooled SMD of −0.06435 with a 95% confidence interval ranging from −0.20447 to 0.07577, encompassing zero. This finding underscores that there were no statistically significant differences in depressive symptoms between the control and experimental groups at the study’s outset. The Chi-squared test for heterogeneity presented a value of 4.98 (degrees of freedom = 10) with a *p*-value of 0.893, indicating negligible heterogeneity across studies. The consistency across studies was further corroborated by a tau-squared (τ^2^) value of 0.

These baseline comparisons confirm the foundational assumption of the experimental design, affirming no significant differences between groups prior to intervention. This critical baseline equivalence supports the further exploration of exercise interventions as a viable strategy for depression prevention in middle-aged and older adults, ensuring that any subsequent evaluation of intervention efficacy is not confounded by pre-intervention disparities.

#### 3.4.2 Meta-analysis result

**Figure 3:**
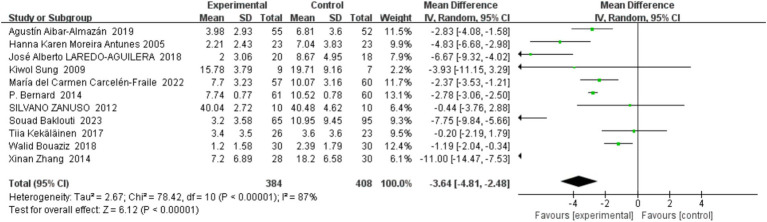
Forest plot of exercise-based prevention of depression in middle-aged and older adults.

This meta-analysis incorporated 11 trials, encompassing a total of 792 middle-aged and older individuals, to assess the impact of exercise on depressive symptoms. The experimental group engaged in regular physical activity, while the control group did not participate in any specific exercise regimen. A random effects model was applied to account for potential heterogeneity among the included studies. The analysis revealed a pooled standardized mean difference (SMD) of −3.64, with a 95% confidence interval ranging from −4.81 to −2.48, as illustrated in Figure 3. This significant reduction in depressive symptoms in the experimental group relative to the control group (*p* < 0.00001) underscores the efficacy of exercise as a preventive strategy against depression among older adults. Notwithstanding the robust overall effect, the I^2^ statistic indicated a high degree of heterogeneity (87%), suggesting considerable variability in effect sizes, potentially due to differences in study design, participant demographics, and the specificities of the exercise interventions.

To probe the sources of this heterogeneity, sensitivity analyses were conducted by sequentially omitting individual studies. These analyses demonstrated minimal changes in heterogeneity, indicating that no single study disproportionately influenced the overall effect size. Further scrutiny suggested that variations in the depressive symptom measures and the focus on specific subpopulations, such as menopausal women, might contribute to the observed discrepancies. The three studies targeting menopausal women highlighted how physiological and psychological shifts during menopause might heighten depression risk, potentially affecting the intervention outcomes. Moreover, the diversity in measurement tools—including the Hamilton Depression Rating Scale (HDRS), Beck Depression Self-Rating Inventory (BDI), and Geriatric Depression Scale (GDS)—each with different sensitivities and specificities, may also amplify heterogeneity.

The findings of this meta-analysis reinforce the protective role of exercise against depression in older adults. The significant evidence advocates for physical activity as a viable preventative approach. However, the pronounced heterogeneity signals a need for future research to adopt more standardized measures of depressive symptoms and to tailor interventions to the unique needs of distinct demographic groups, such as menopausal women, enhancing the generalizability and effectiveness of exercise-based prevention strategies.

#### 3.4.3 Publication bias test

In our meta-analysis, a sensitivity analysis was performed using the Leave-One-Out Analysis approach to assess the robustness of our findings. By sequentially removing each study and recalculating the pooled effect size, we found that the overall heterogeneity did not change significantly, indicating that no single study disproportionately influenced the results. This suggests that our findings are stable and not driven by any particular study.

**Figure 4:**
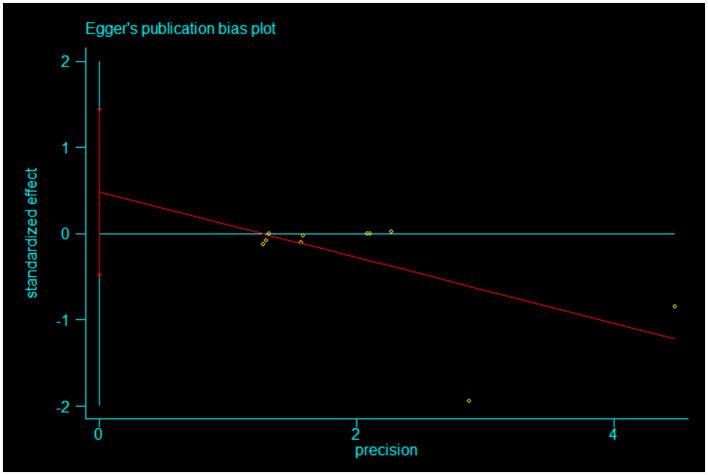
Egger’s publication bias plot.

In our meta-analysis, a critical evaluation of publication bias was conducted using Egger’s test, as depicted in Egger’s publication bias plot (Figure 4). This choice was guided by several considerations: Egger’s test is a quantitative method adept at detecting small study effects and evaluating publication bias, especially suitable for analyses involving continuous variables. It examines the association between effect sizes and their statistical precision via regression analysis, making it an effective tool for identifying possible correlations in studies with continuous data outcomes.

Despite the availability of various methods for assessing publication bias, such as Begg’s test or visual inspection of funnel plots, Egger’s test was deemed more sensitive for our dataset, which includes a number of small studies. This sensitivity is crucial for accurately detecting bias in smaller samples. While acknowledging the limitations of Egger’s test, including its reduced sensitivity to nonlinear effect size alterations and potential inaccuracies under extreme heterogeneity, its applicability was confirmed after a thorough review of our data and study design specifics.

The results of the Egger’s test indicated a slope coefficient of −0.3796654 (standard error 0.1816327), yielding a *t*-value of −2.09 and a *p*-value of 0.070. Although this result approached the conventional threshold of statistical significance (*p* < 0.05), it did not achieve it, suggesting a potential but not definitive correlation between effect size and standard error. Furthermore, the 95% confidence interval for the slope coefficient, ranging from −0.7985111 to 0.0391802, underscores the instability of this trend.

**Figure 5:**
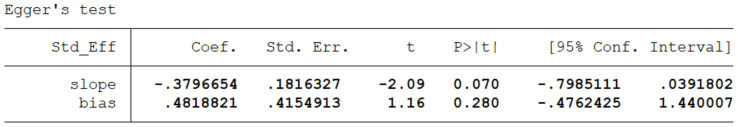
Egger’s test.

Additionally, the bias coefficient was 0.4818821 (standard error 0.4154913) with a t-value of 1.16 and a *p*-value of 0.280; the 95% confidence interval ranged from −0.4762425 to 1.440007. These results, shown in Figure 5, indicate no statistically significant bias.

Although Egger’s test did not conclusively reveal significant publication bias, the proximity of the *p*-value to the significance level warrants a prudent approach in interpreting these findings. The absence of detectable significant publication bias lends credibility to the meta-analysis results but also highlights the necessity for ongoing scrutiny, particularly in studies with smaller sample sizes, to ensure robust and reliable conclusions in future research.

#### 3.4.4 Subgroup analysis

In this study, we meticulously examined subgroups based on the modality and duration of exercise interventions, as well as whether exercises were performed individually or in groups. Exercise types were systematically divided into four specific interventions: aerobic training, resistance training, balance and flexibility training, and functional training. The duration of individual exercise sessions was categorized into three distinct groups: short-duration exercise ranging from 30 to 40 min, medium-duration exercise lasting approximately 60 min, and long-duration exercise extending to 80 min.

Additionally, the overall duration of the exercise interventions was segmented into three subgroups to assess the impact over varying periods: short-term (less than 3 months), medium-term (3 to 6 months), and long-term (more than 6 months). These classifications facilitate a structured analysis of how different exercise durations and types influence health outcomes, allowing for a nuanced understanding of the most effective exercise regimens for specific populations or health objectives.

##### 3.4.4.1 Individual versus group

In this meta-analysis, we applied a random effects model to address potential heterogeneity among the included trials, which were divided into two subgroups: group exercise interventions and individual exercise interventions. Our objective was to evaluate the differential impacts of these intervention types on the prevention of depression in middle-aged and older adults.

**Figure 6:**
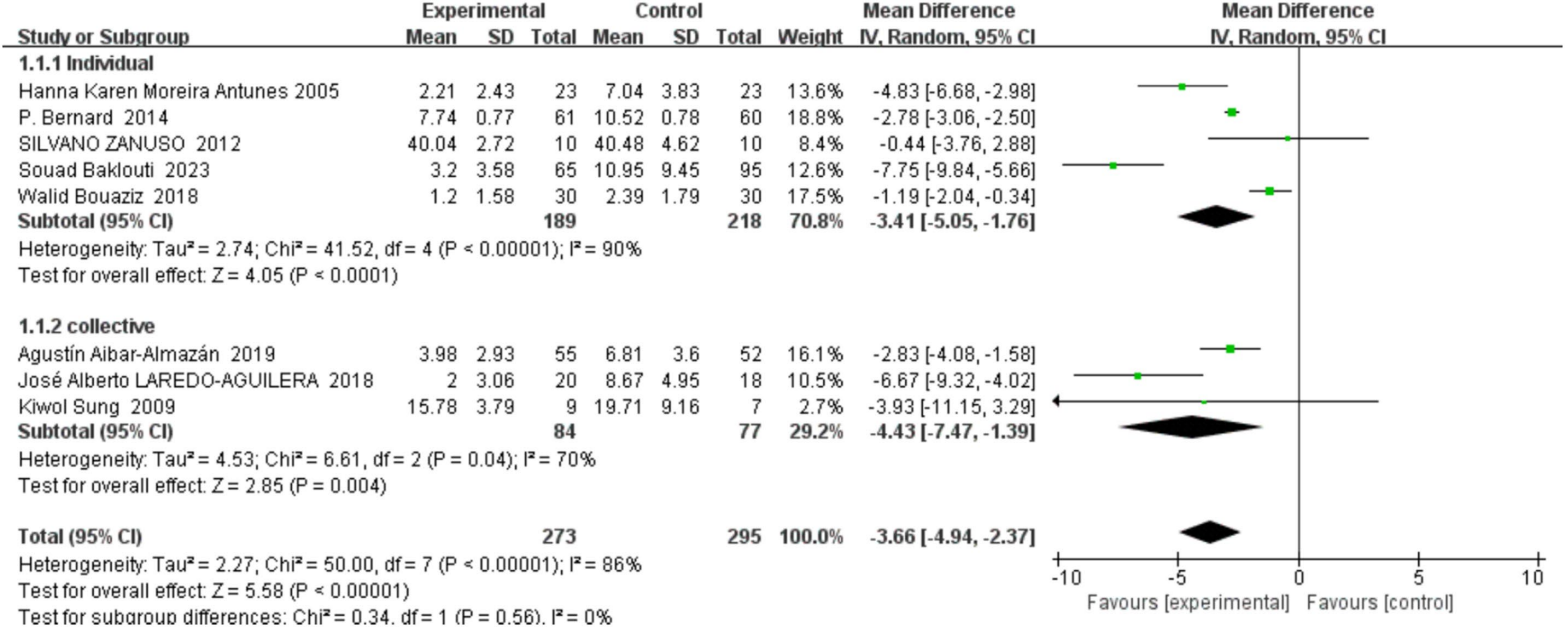
Forest plot of subgroup analyses by individual versus group.

For the group exercise interventions, heterogeneity was relatively low (*I*^2^ = 70%), and the overall effect size indicated a significant antidepressant effect (Standardized Mean Difference, SMD = −4.43, 95% Confidence Interval, CI [−7.47, −1.39]). The individual intervention subgroup, however, exhibited higher heterogeneity (*I*^2^ = 90%) with an overall effect size (SMD = −3.41, CI [−5.05, −1.76]) also demonstrating effectiveness in mitigating depressive symptoms. These subgroup findings are detailed in Figure 6. Analysis of subgroup differences showed no significant disparity between the individual and group interventions (Chi^2^ = 0.34, degrees of freedom = 1, *p* = 0.56), indicating that the modality of exercise delivery—whether group or individual—does not significantly affect the intervention’s efficacy in depression prevention among this demographic. The overarching analysis underscored a significant positive impact of exercise interventions overall (SMD = −3.66, CI [−4.94, −2.37]), reinforcing the role of physical activity as a beneficial measure for middle-aged and older individuals at risk of depression. This outcome aligns with prior research advocating for exercise as a preventive strategy against depression in older adults.

Despite a slight numerical superiority in the effect size of group interventions over individual ones, the lack of statistical significance between the subgroup effects suggests that both modalities are comparably effective. The observed differences may stem from variations in sample sizes, study designs, and the specifics of the exercise regimens, such as duration and intensity. Therefore, our findings suggest that both group and individual exercise formats can be equally effective in preventing depression among middle-aged and older adults, highlighting the flexibility of intervention strategies that can be tailored to diverse population needs and preferences.

##### 3.4.4.2 Type of exercise

**Figure 7:**
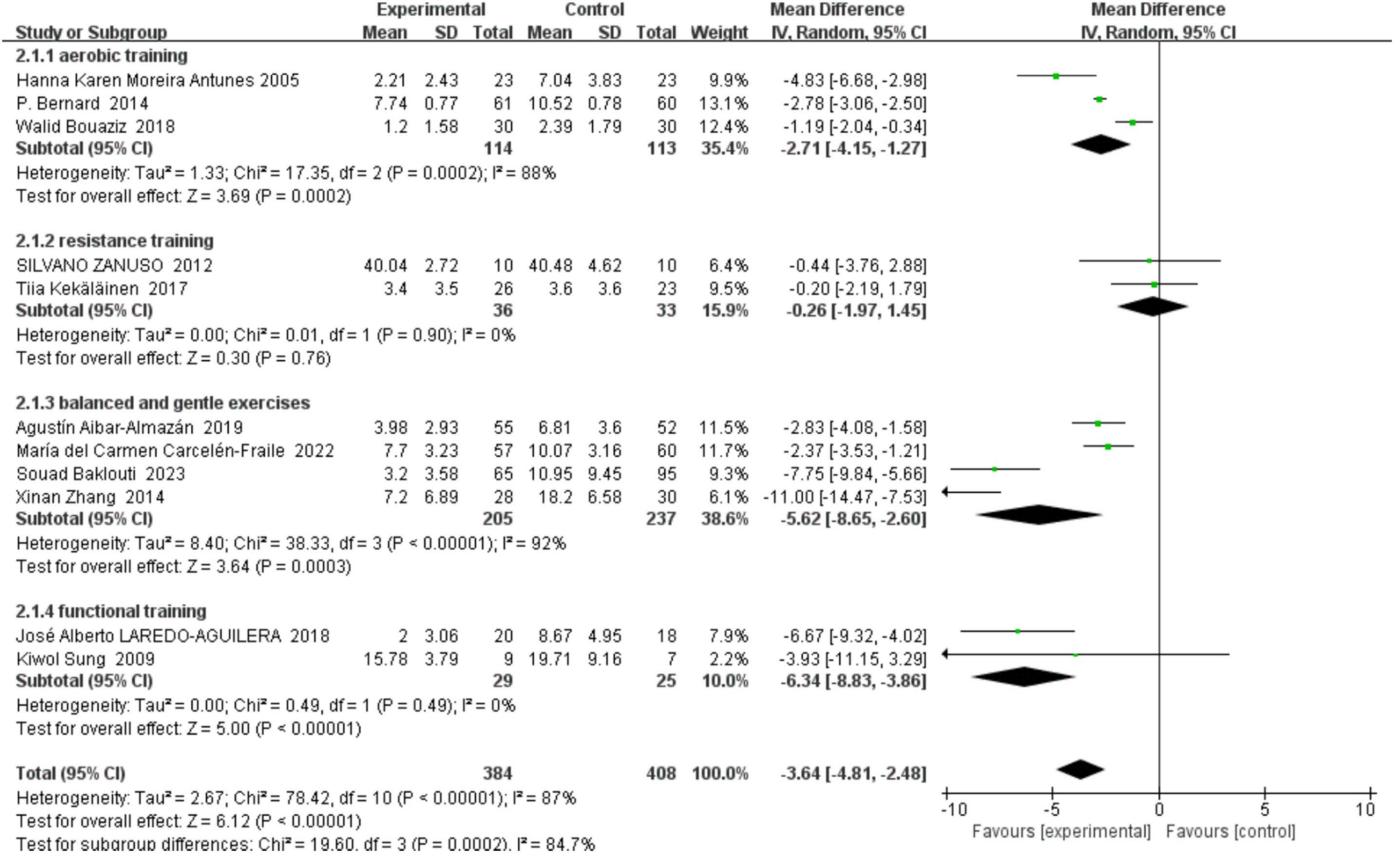
Forest plot of subgroup analyses by type of exercise.

The imperative for conducting subgroup analyses in systematic reviews and meta-analyses stems from the nuanced effects that different types of exercise interventions may exert on depression prevention among middle-aged and older adults. Given the heterogeneity in physical conditions, exercise preferences, and psychological states within this demographic, a singular analytical approach might not adequately capture the specific impacts of each exercise modality on health outcomes. Consequently, we categorized exercise interventions into four distinct subgroups: ‘aerobic training,’ ‘resistance training,’ ‘balance and flexibility training,’ and ‘functional training.’ This classification enables a deeper understanding of which exercise types are most effective at mitigating depressive symptoms, detailed in Figure 7.

###### 3.4.4.2.1 Subgroup analysis findings

Aerobic Exercise: Involving studies such as those by Hanna Karen Moreira Antunes (2005) and Walid Bouaziz (2018), this subgroup analysis revealed that aerobic exercise significantly reduced depressive symptoms in middle-aged and older adults with a mean difference of −2.71 (95% CI: −4.15, −1.27, *p* < 0.0002). However, the high heterogeneity (*I*^2^ = 88%) suggests variability potentially due to participant baseline differences or specific exercise modalities employed across studies.

Resistance Training: Including studies like Silvano Zanuso (2012) and Tiia Kekäläinen (2017), resistance training showed no significant effect on depressive symptoms prevention (mean difference: −0.26, 95% CI: −1.97, 1.45, *p* = 0.76). The very low heterogeneity (*I*^2^ = 0%) indicates a consistent lack of effect across different studies.

Balance and Flexibility Training: Reviewed studies such as those by Agustin Albar-Almazán (2019), María del Carmen Carcelén-Fraile (2022), and Xinan Zhang (2014) focused on gentle modalities like Tai Chi. This type of training significantly prevented depressive symptoms (mean difference: −5.62, 95% CI: −8.65, −2.60, *p* < 0.00003), albeit with extremely high heterogeneity (*I*^2^ = 92%), suggesting significant variability in effects among the studies.

Functional Training: Studies such as those by José Alberto Laredo-Aguilera (2018) and Kiwol Sung (2009) demonstrated that functional training significantly prevents depressive symptoms (mean difference: −6.34, 95% CI: −8.83, −3.86, *p* < 0.00001) with very low heterogeneity (*I*^2^ = 0%), indicating a consistent effect across studies.

By analyzing these subgroups, it becomes apparent that balance and gentle training, as well as functional training, are particularly effective in preventing depressive symptoms in the targeted demographic, with aerobic training also showing benefits. Despite resistance training not displaying a significant effect, its high consistency across studies offers valuable insights for clinical application. These findings emphasize the importance of customizing and optimizing exercise programs for middle-aged and older adult individuals at risk of depression through detailed subgroup analysis. Future research should delve into the physiological and psychological mechanisms underpinning these differential effects and how exercise protocols can be best tailored to accommodate individual variations.

##### 3.4.4.3 Exercise session lengths

**Figure 8:**
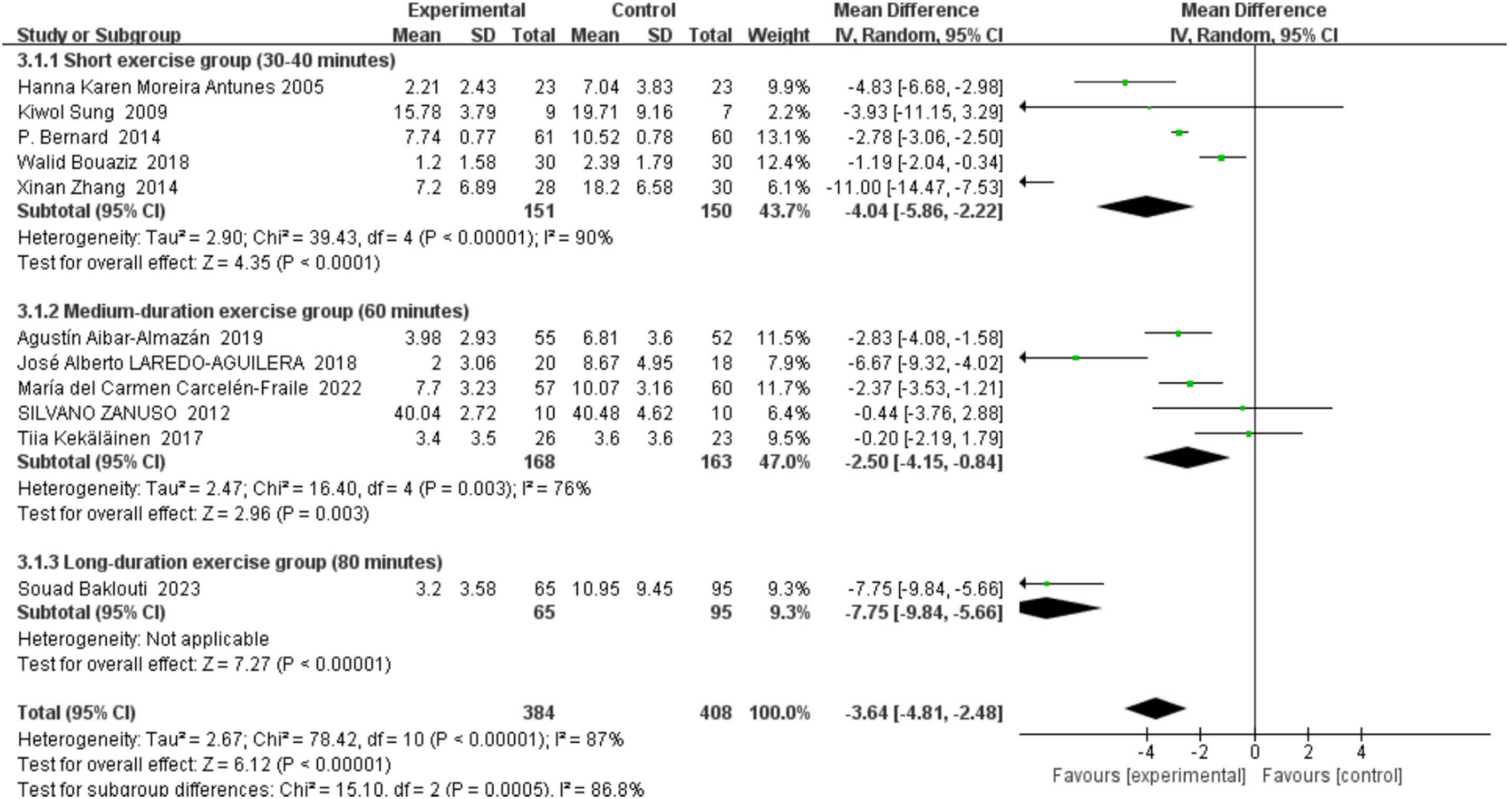
Forest plot of subgroup analyses by exercise session lengths.

In this systematic review and meta-analysis, we meticulously explored the potential therapeutic effects of varying exercise session durations on depression prevention in middle-aged and older adults. Given the potential impact of exercise duration on health outcomes, we stratified the included studies into three subgroups based on session length: ‘short-term exercise group’ (30–40 min), ‘medium-term exercise group’ (60 min), and ‘long-term exercise group’ (80 min), with detailed results depicted in Figure 8.

###### 3.4.4.3.1 Short-term exercise group (30–40 min)

This subgroup includes studies from Hanna Karen Moreira Antunes (2005), Kiwol Sung (2009), P. Bernard (2014), Walid Bouaziz (2018), and Xinan Zhang (2014). The combined results indicated a significant preventive effect on depressive symptoms, with a mean difference of −4.04 (95% CI: −5.86, −2.22), *p* < 0.0001. High heterogeneity (*I*^2^ = 90%) within this group suggests variability likely due to the diversity in exercise types and participant baseline status.

###### 3.4.4.3.2 Intermediate exercise group (60 min)

This category comprised studies by Agustin Albar-Almazán (2019), José Alberto Laredo-Aguilera (2018), María del Carmen Carcelén-Fraile (2022), Silvano Zanuso (2012), and Tiia Kekäläinen (2017). Analysis showed a mean difference of −2.50 (95% CI: −4.15, −0.84), *p* = 0.003, indicating that moderate-duration exercise effectively prevents depressive symptoms. The relatively low heterogeneity (*I*^2^ = 76%) suggests a good level of consistency among the included studies.

###### 3.4.4.3.3 Long-term exercise group (80 min)

Only one study, by Souad Baklouti (2023), fell into this subgroup, showing a significant reduction in depressive symptoms with a mean difference of −7.75 (95% CI: −9.84, −5.66), *p* < 0.00001. As this subgroup contained only one study, heterogeneity could not be assessed, making the results less conclusive.

Overall, our findings demonstrate that different durations of exercise sessions significantly reduce depressive symptoms in middle-aged and older adults. Long-term exercise displayed the most robust effect; however, the strength of this evidence is limited due to the inclusion of a single study. Both short- and medium-term exercises also exhibited beneficial effects, with short-term exercise appearing particularly effective, albeit with varied heterogeneity reflecting the diversity in study designs and exercise modalities. These insights underscore the importance of tailoring exercise programs to individual needs to optimize the prevention of depression, suggesting that personalized approaches may enhance therapeutic outcomes.

##### 3.4.4.4 Duration of the exercise programmes

**Figure 9:**
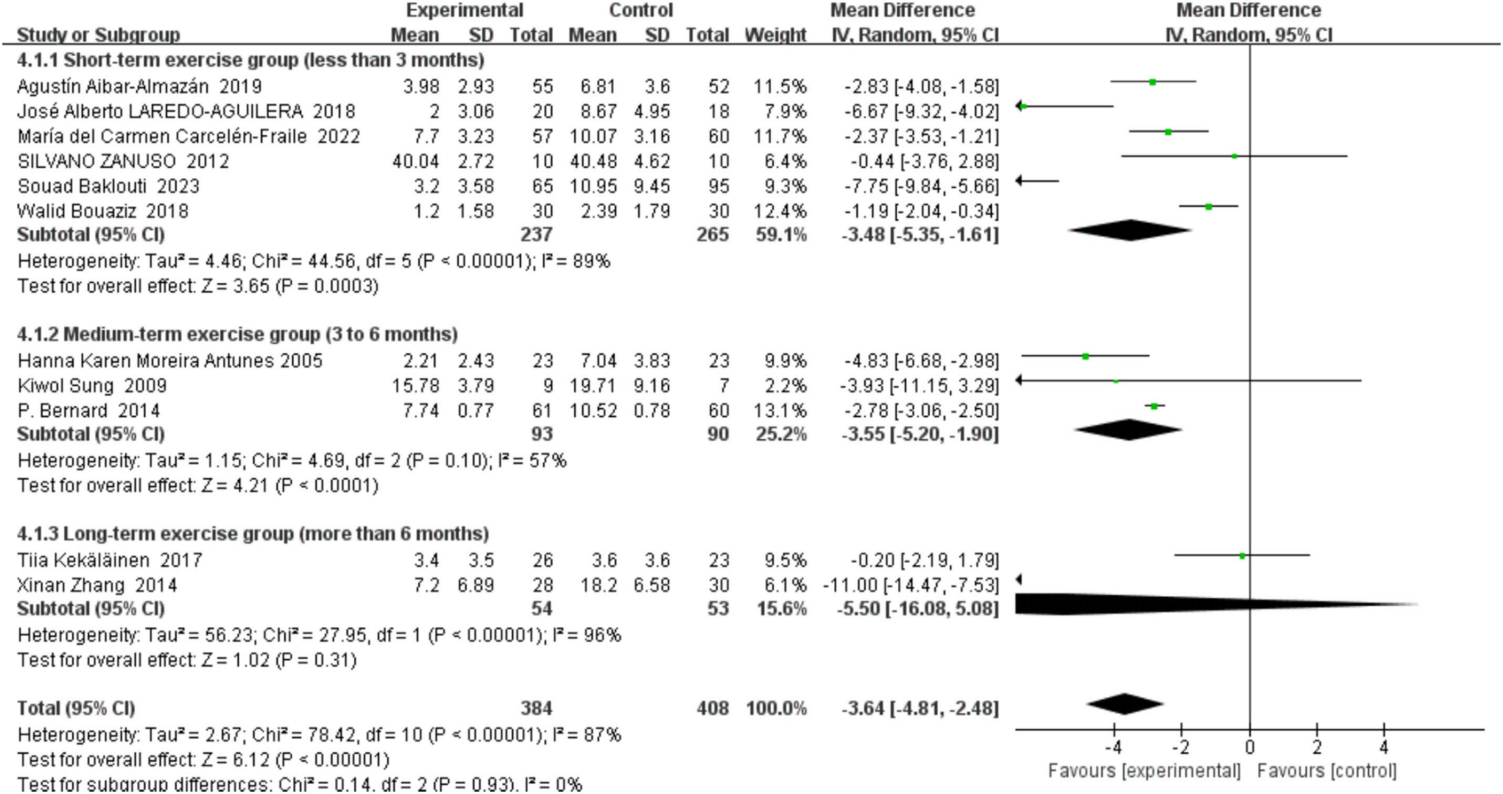
Forest plot of subgroup analyses by exercise session lengths.

In conducting this systematic review and meta-analysis, we placed a significant emphasis on subgroup analyses concerning the total duration of exercise interventions, as this parameter can critically influence the prevention of depressive symptoms in middle-aged and older adults. The studies included in our review were classified into three duration-based subgroups: short-term (less than 3 months), intermediate-term (3 to 6 months), and long-term (more than 6 months). This stratification enabled a detailed evaluation of how varying lengths of exercise interventions impact depressive symptom prevention, with findings detailed in Figure 9.

Short-term exercise group (less than 3 months): This subgroup includes studies by Agustin Albar-Almazán (2019), José Alberto Laredo-Aguilera (2018), María del Carmen Carcelén-Fraile (2022), Silvano Zanuso (2012), Souad Baklouti (2023), and Walid Bouaziz (2018). Collectively, these studies demonstrate a significant preventative effect of short-term exercise on depressive symptoms, with a mean difference of −3.48 (95% CI: −5.35, −1.61, *p* < 0.0001). However, the high heterogeneity (*I*^2^ = 89%) among these studies likely reflects variations in exercise methodologies and participant baseline characteristics.

Intermediate exercise group (3–6 months): This group comprises studies by Hanna Karen Moreira Antunes (2005), Kiwol Sung (2009), and P. Bernard (2014). The results indicate that intermediate-term exercise significantly prevents depressive symptoms, with a mean difference of −3.55 (95% CI: −5.20, −1.90, *p* < 0.0001). The relatively low heterogeneity (*I*^2^ = 57%) suggests a consistent effect across studies, underscoring the effectiveness of medium-duration exercise programs.

Long-term exercise group (more than 6 months): This subgroup includes studies by Tilia Kekäläinen (2017) and Xinan Zhang (2014). The effect of long-term exercise on depressive symptoms, while showing a mean difference of −5.50 (95% CI: −16.08, 5.08, *p* = 0.31), did not reach statistical significance, and exhibited extremely high heterogeneity (*I*^2^ = 96%). This may be attributed to large variations in sample sizes, intensity of exercise interventions, and types of exercise employed in these studies.

Overall, our meta-analysis revealed that exercise interventions of various durations generally exert a positive effect on preventing depressive symptoms in middle-aged and older adults. Notably, while short- and intermediate-term exercises demonstrated significant benefits, the impact of long-term exercise was not statistically significant, potentially due to intrinsic study limitations and high variability among the included studies. This suggests that while moderate-intensity exercise seems to offer slightly better outcomes, the effectiveness of exercise interventions may not necessarily increase with longer durations. These insights highlight the need for tailored exercise programs that consider both the duration and intensity of activity to maximize preventative effects against depression in older adults.

## 4 Discussion

The findings of this meta-analysis underscore the efficacy of exercise as a non-pharmacological intervention for the prevention of depression in middle-aged and older adults. Despite the moderate effect size observed across the studies, substantial heterogeneity was noted, which may be attributed to variations in depression assessment scales, sample sizes, intervention types, and participant demographics. To refine exercise recommendations further, future research should focus on determining the optimal duration, frequency, and intensity of exercise interventions and explore how different exercise modalities can specifically prevent depressive symptoms.

Our systematic search across four databases revealed that both individual and group exercise interventions hold promise for mitigating depression among the target demographic. Although the standardized mean difference indicated a slightly higher efficacy for group exercises, no statistically significant difference was found between group and individual interventions in subgroup analyses. This suggests that while group exercises may offer marginally better outcomes, the evidence does not conclusively favor one mode of delivery over the other. Such findings highlight the influence of variable factors including study design, sample size, and intervention specifics on the results. Consequently, we conclude that both individual and group exercise interventions effectively prevent depression in middle-aged and older adults, providing similar benefits. This reinforces the value of integrating exercise into preventive health strategies for this population, irrespective of the social setting of the activity.

The differential impact of various exercise modalities on the prevention of depressive symptoms in middle-aged and older adults is a critical finding from our meta-analysis. Balance and gentleness training, as well as functional training, have demonstrated significant efficacy in mitigating depressive symptoms in this demographic. Although aerobic training also offers benefits, it is somewhat less effective than these modalities. Resistance training, despite its lack of significant impact, showed remarkable consistency across studies, providing valuable insights for clinical application. The observed effectiveness of functional and balance training in mitigating depressive symptoms may be attributed to several interrelated physiological and psychological mechanisms. First, these modalities are known to enhance neuroplasticity, promoting structural and functional adaptations in brain regions implicated in mood regulation, such as the hippocampus and prefrontal cortex. Regular engagement in such training has been shown to upregulate brain-derived neurotrophic factor (BDNF), a key mediator of synaptic plasticity and neuronal resilience, which is often reduced in individuals with depression. Second, the inherently social nature of functional training, particularly in group-based settings, may contribute to its superior effects. Social interaction during structured exercise fosters a sense of belonging, reduces perceived stress, and modulates the hypothalamic–pituitary–adrenal (HPA) axis, potentially mitigating the neuroendocrine dysregulation associated with depression (26). Lastly, the cognitive demands of balance and functional exercises, which require coordination, executive function, and sensorimotor integration, may provide additional neurocognitive stimulation. Emerging evidence suggests that such activities engage frontostriatal circuits, which are implicated in both motor control and affective regulation, further reinforcing their antidepressant effects (27). Collectively, these findings suggest that functional and balance training may exert a multidimensional impact on mental health, underscoring the need for future studies to elucidate the precise neurobiological pathways underlying these effects.

Our analysis further emphasizes the importance of customizing exercise programs to individual needs, enhancing the effectiveness of interventions aimed at preventing depression (28). From a physiological perspective, all forms of exercise can combat depressive symptoms in older adults through several mechanisms, including the modulation of endocrine functions (29). These mechanisms involve elevating neurotrophic factors, enhancing adipocytokine production, regulating neurotransmitter expression, improving mitochondrial function, boosting melatonin secretion, reducing inflammation, and influencing the expression of microRNAs (14, 30, 31).

Specific exercise interventions yield unique benefits due to their distinct physiological and psychological impacts (32). For instance, balance and gentleness exercises such as tai chi focus more on psychological relaxation and physical balance, whereas aerobic exercises enhance cardiorespiratory fitness and overall endurance (33, 34). However, the suitability of high-intensity aerobic activities may be limited for some older adults due to age-related physical constraints.

Therefore, it is crucial to consider individual differences when designing exercise programs. Older adults with poorer physical health or specific medical conditions might be better suited to low-intensity, functional training, which is less likely to impose undue stress (35). In contrast, healthier older individuals might gain more from the rigorous physical demands of aerobic training.

To prevent depressive symptoms effectively, it is essential to select an exercise regimen that is scientifically sound and tailored to the individual’s health status and capabilities. This approach ensures that exercise serves as a beneficial tool rather than a source of additional stress, optimizing mental and physical health outcomes for middle-aged and older adults (36). This tailored approach underscores the need for a nuanced understanding of the interactions between different exercise types and individual health profiles in the aging population.

Subgroup analyses in our study reveal that varying durations of exercise significantly mitigate depressive symptoms in middle-aged and older adults, although long-term exercise displayed the most pronounced effect. However, this finding is tempered by the inclusion of only a single study for long-term exercise, limiting the robustness of this conclusion. In contrast, both short-term and intermediate-term exercises demonstrated considerable therapeutic benefits, with short-term exercise emerging as particularly effective despite varied heterogeneity, which likely reflects diverse study designs.

These results underscore the necessity of crafting individualized exercise programs tailored to optimize the prevention of depression. Future research should strive to control for the potential sources of heterogeneity identified in our analyses to provide more precise recommendations regarding the most effective exercise durations for improving symptoms in middle-aged and older adults. Notably, while short-term exercise may effectively address certain physiological and psychological states, long-term exercise could offer more sustained benefits in areas such as cardiovascular health and psychological well-being.

Our findings indicate a generally positive impact of exercise on depressive symptoms across different exercise durations, although the statistical significance of long-term exercise’s effects remains inconclusive, possibly due to study limitations and high heterogeneity. Interestingly, medium-term exercise exhibited a slightly superior effect compared to short-term regimens, suggesting that duration may play a critical role in optimizing exercise benefits.

This study solidifies the role of exercise interventions in preventing depressive symptoms among middle-aged and older adults, providing a strong foundation for applying these insights in clinical practice and public health policy. For practical implementation, healthcare professionals should conduct thorough initial assessments, considering individuals’ baseline physical fitness, comorbidities, and psychological status to customize exercise programs that optimize adherence and effectiveness. Given the strong evidence supporting moderate-duration (30–40 min) group-based functional training, these modalities should be prioritized when designing interventions for this demographic.

From a policy perspective, integrating structured exercise interventions into existing public health programs—such as community health centers, senior wellness programs, and primary care initiatives—could enhance accessibility and participation. Governments and health organizations should allocate funding and infrastructure to support instructor-led exercise programs, particularly for underserved populations, ensuring equity in mental health interventions. Additionally, public awareness campaigns highlighting the mental health benefits of exercise, along with incentives such as subsidized gym memberships or tax benefits for participation in structured exercise programs, could further promote adherence and engagement.

Finally, collaboration between public health agencies, community organizations, and healthcare providers is essential for scaling up effective interventions and embedding them into routine preventive healthcare. Establishing standardized guidelines on exercise prescription for mental health, based on evidence from systematic reviews and meta-analyses, would provide clearer recommendations for practitioners and decision-makers. Through these targeted strategies, exercise can be effectively leveraged as a non-pharmacological, accessible, and sustainable approach to improving mental well-being in aging populations.

Despite the compelling evidence presented in this meta-analysis, several limitations must be acknowledged. The high heterogeneity across studies (*I*^2^ = 87%) indicates considerable variability in intervention types, participant characteristics, and outcome measures, which may affect the precision of the pooled estimates. While subgroup analyses were conducted, residual heterogeneity suggests that additional factors—such as baseline health conditions, socioeconomic status, and cultural differences—may influence the effectiveness of exercise interventions in preventing depression. Moreover, the total sample size of 792 subjects, though meaningful, may limit the generalizability of our findings to broader and more diverse populations. Many of the included studies had relatively small sample sizes and varying methodological quality, raising concerns about potential biases in effect estimates.

Additionally, our subgroup analysis on exercise duration suggests that moderate-duration exercise (30–40 min per session) is the most effective, yet the observed benefit of long-duration exercise (>80 min) is derived from a single study, limiting its interpretability. Given this constraint, caution is warranted in drawing conclusions about the efficacy of prolonged exercise sessions, and future research should aim to replicate these findings with larger, more diverse samples. Furthermore, the lack of an in-depth exploration of moderating factors, such as comorbidities and psychosocial influences, constrains our understanding of the specific conditions under which exercise exerts its strongest protective effects. Finally, while our findings highlight the promise of exercise as a preventive strategy against depression, they underscore the need for larger, more rigorously designed randomized controlled trials to validate these effects and establish optimal intervention protocols.

## 5 Conclusion

Our study conclusively identifies several key facets of exercise as a preventive measure against depression in middle-aged individuals. We delineate the efficacy of different exercise modalities, the optimal duration of exercise sessions, and the overall length of exercise interventions. Herein, we highlight the notable distinctions between individual and group exercise interventions in mitigating depressive symptoms.

Effectiveness of Exercise Modalities: We discovered that balance-oriented and gentle training, as well as functional training, significantly thwart the onset of depression among middle-aged adults. Optimal Exercise Session Duration: Our findings suggest that shorter exercise sessions, lasting between 30 and 40 min, are most effective. Intervention Duration: Medium-term exercise regimens slightly outperform shorter-term interventions, indicating a nuanced response over different intervention lengths. Group vs. Individual Exercise: Group exercise settings proved more beneficial than solitary sessions, highlighting the potential importance of social interaction in preventive strategies.

Directions for Future Research: Despite the progress detailed in this study, several research avenues remain uncharted. Key among them is the need to unravel the specific mechanisms at the physiological and psychological levels through which exercise exerts its antidepressant effects. This understanding is crucial for refining and optimizing intervention protocols. Furthermore, the heterogeneity observed in the current literature—primarily stemming from a focus on short-term interventions—necessitates a thorough investigation into the long-term effects of sustained exercise. Most extant studies concentrate on immediate outcomes, leaving a gap in our understanding of the enduring benefits of regular physical activity. Lastly, it is imperative to explore how different demographic factors such as gender, cultural background, and socioeconomic status influence the efficacy of exercise interventions. Such differentiated insights will pave the way for more tailored and effective preventive measures against depression in diverse populations of middle-aged and older adults.

## Data Availability

Publicly available datasets were analyzed in this study. This data can be found here: since this study is a meta-analysis, the data used for the analysis were extracted from previously published studies. A comprehensive search was conducted across several databases, including PubMed, Embase, Web of Science, and the Cochrane Library, and all relevant studies were included based on predefined eligibility criteria. As the data are secondary and publicly available through these sources, there is no direct dataset associated with this manuscript.
