# Supplementary material for: Move your body, stay away from depression: a systematic review and meta-analysis of exercise-based prevention of depression in middle-aged and older adults
Source: Front Public Health. 2025 Mar 7;13:1554195. doi: 10.3389/fpubh.2025.1554195 (PMC11925714; doi:10.3389/fpubh.2025.1554195)
Supplement: Supplementary file 2 [file Presentation_2.pdf]

	Random sequence generation (selection bias)	Allocation concealment (selection bias)	Blinding of participants and personnel (performance bias)	Blinding of outcome assessment (detection bias)	Incomplete outcome data (attrition bias)	Selective reporting (reporting bias)	Other bias
Agustín Aibar-Almazán 2019	+	+	−	+	+	+	?
Hanna Karen Moreira Antunes 2005	+	?	−	?	+	+	?
José Alberto LAREDO-AGUILERA 2018	+	?	?	+	+	+	?
Kiwol Sung 2009	+	+	−	?	+	+	?
María del Carmen Carcelén-Fraile 2022	+	+	−	?	+	+	?
P. Bernard 2014	+	+	−	?	+	+	?
SILVANO ZANUSO 2012	+	?	−	?	+	+	?
Souad Baklouti 2023	+	+	−	?	+	+	?
Tiia Kekäläinen 2017	+	+	−	?	+	+	?
Walid Bouaziz 2018	+	+	−	+	+	+	?
Xinan Zhang 2014	+	?	−	?	+	?	?
